# Supplementary material for: High Frequency of PIK3CA Mutations in Low-Grade Serous Ovarian Carcinomas of Japanese Patients
Source: Diagnostics (Basel). 2019 Dec 27;10(1):13. doi: 10.3390/diagnostics10010013 (PMC7168240; doi:10.3390/diagnostics10010013)
Supplement: Supplementary file 1 [file diagnostics-10-00013-s001.pdf]

Supplementary Table S1. Primer sequence of mutation analysis.

KRAS-Exon2-Forward	TTAACCTTATGTGTGACATGTTCTAA
KRAS-Exon2-Reverse	AGAATGGTCCTGCACCAGTAA
BRAF-Exon15-Forward	TGCTTGCTCTGATAGGAAAATG
BRAF-Exon15-Reverse	AGCATCTCAGGGCCAAAAAT
PIK3CA-Exon9-Forward	GGGAAAAATATGACAAAGAAAGC
PIK3CA-Exon9-Reverse	CTGAGATCAGCCAAATTCAGTT
PIK3CA-Exon20-Forward	CTCAATGATGCTTGGCTCTG
PIK3CA-Exon20-Reverse	TGGAATCCAGAGTGAGCTTTC
ERBR2-Exon20-Forward	CCATACCCTCTCAGCGTAC
ERBR2-Exon20-Reverse	CGGAGAGACCTGCAAAGAG

Supplementary Table S2. 01 Mutational analysis of Low grade serous carcinoma in both Japan and Western countries

**Author's Name**	***KRAS***	**BRAF**	**PIK3CA**	**ERBB2**	**Journal, Year**	**Race. (Country)**	**References**
Wong K.K. et. al.	19%	2%			Am. J. Pathol. 2010	USA	3
Jones, S. et. al.	19%	38%	4%		J. Pathol. 2012	USA	4
Singer, G. et. al.	54%				Am. J. Pathol. 2002	USA	5
Nakayama, K. et. al.	33%	24%	5%	9.5%	Cancer Biol. Ther. 2006	USA	7
Xu, Y. et. al.	28%	6%			Diagn. Pathol. 2012	Chinese	11
Nieuwenhuysen, E.V. et al.	18.40%	10.50%	5.20%	0%	Neoplasia. 2019	Belgium	12
Hunter, S.M. et al.	21.00%	16.00%		0%	Oncotarget 2015	Australia, Canada	13
Current Studat	0%	20%	60%	30%	2019	Japanese	
